# Book Reviews

**Published:** 1822-09

**Authors:** 


MONTHLY

### MEDICAL AND SCIENTIFIC BIBLIOGRAPHY.

Vexat censura . . . . . . CORVOS.

ART. I.—*Cases in Surgery*. By HENRY JEFFREYS. 8vo. pp. 237.

THIS volume, which is dedicated to the governors of the St. James's and St. George's Dispensary, of which the author is senior surgeon, contains the first fruits of his practice at that charity. Its contents are of a miscellaneous nature; they are treated in a perspicuous and unaffected manner as to style and composition, and afford abundant evidence of the zeal and ability of the writer. The subjects treated of are Strumous Ophthalmia; the Effects of Tartar Emetic in subduing Inflammation; on the Treatment of Mammary Abscess; and the volume concludes with Observations on the Employment of Elm-Bark as a Substitute for Sarsaparilla. There are also two or three single cases detailed.

We have nothing to observe upon the first of these subjects. Mr. Jeffreys has given an accurate description of this complaint, so commonly met with among the poor of this metropolis: his practice is unobjectionable, and numerous cases in illustration are detailed. In treating on the effects of the internal exhibition of tartar emetic, he very candidly ascribes to Dr. BALFOUR the merit of having directed the attention of the profession to the efficacy of this medicine as a powerful sedative, and as dispensing, in many instances, with the necessity of blood-letting to so great an extent as is often practised. We concur in these sentiments generally, and are ready to join our testimony to that of our author as to the great utility of the medicine in hernia humoralis, in particular; and we think he has avoided the too common fault of over-praising a new, or at least a newly applied, remedy. The preparation usually employed by Mr. Jeffreys was the following:

℞ Antimon. Tartar. gr. iij.

Magnes. Sulphat. ℥j.

Aq. Fontanæ, ℥vj. M. ft. Mist.

Two table-spoonsful of this mixture was usually ordered to be taken three times a-day; though, according to the urgency of symptoms, it was occasionally given in larger doses, and more frequently.

We shall merely notice the cases of mammary abscess, for the purpose of remarking that the plan of strapping the breast after the abscess has broke and the slough is thrown off, though a good one, must not be had recourse to too soon; nor should the pressure made be great, or to such a degree as to produce any uneasy feeling in the part. We recollect the above plan to have been recommended by our much esteemed teacher, Mr. Pearson.

The remarks upon the use of Elm-Bark are of great importance to those surgeons, especially, who are attached to charitable institutions, as the expence is trifling in comparison with that of sarsaparilla; and we can speak decidedly as to the benefits derived from its employment in most of those anomalous cases, for the cure of which that medicine would have been prescribed. We shall conclude by subjoining the formula for the decoction, as employed by our author:

℞ Decocti Ulmi (Ph. L.) oc. viij.

Sassafras rud. concisæ,

Guaici Ligni rasi sing. ℥j.

Mezer. rad. Corticis, ʒiij.

Glycyrrhisæ rad. Contusæ, ℥j.

Decoque per horam et cola.

The dose is from half a pint to one pint in the day.

---

Art. II.—*Researches respecting the Medical Powers of Chlorine, particularly in Diseases of the Liver.* By W. Wallace, M.R.I.A. 8vo. pp. 148.

We are sorry that Mr. Wallace felt it necessary to publish this work before he was prepared with the plate descriptive of the apparatus made use of in applying the chlorine to the surface of the body generally; since what he says of its effects render us most anxious to make trial of it; and, in consequence of that omission, we are under the necessity of repressing our curiosity, and must limit our operations to the humble task of detailing to our readers the beneficial results our author has derived from its employment.

We confess it appears to us that the facts illustrative of the utility of this remedy might have been comprised much within the compass of 148 pages; and our conviction became stronger from remarking the mode in which the cases are detailed: they are infinitely too long, and encumbered with too many explanations, and too much speculative reasoning. We cannot help thinking that it is always much better to suffer readers to draw their own conclusions from a simple detail of facts; a practice sanctioned by the authority of some of the brightest ornaments of our profession. In illustration of what we assert, we shall quote a remark or two of the author's at the conclusion of his first case, which is one of jaundice combined with affection of the liver, and which occupies six pages of the work. The subject was a lad, and the facts simply these:—He had been ill for six weeks; had been at several hospitals; and had taken purgatives and other remedies, the nature of which he (Mr. Wallace) could not ascertain; he was subjected to treatment on the 14th of May, on the evening of which day he took ten grains of extr. colocynth comp., and four drachms of sulphate of magnesia the following morning. On the 15th, his body was exposed to the chlorine; and, not being able to remain longer in town than the 24th, he was permitted to go into the country, taking with him some of his purgative pills and salts; and, being at that time convalescent, at the end of five weeks he called on Mr. Wallace, perfectly restored in all respects. Mr. W. here congratulates himself upon the result of this case, and thinks that the removal of the disease could only be owing to the chlorine, which, it must be observed, was employed but eight times, whilst the purgatives were occasionally administered during five weeks. This surely will not be quite so convincing to others as it appears to have been to our author; but the following remark is still less satisfactory:—"Although I cannot speak positively," says Mr. W. "yet I think there are but few who will not be disposed to agree in opinion with me, that it is more than probable,—indeed, that it can scarcely be doubted,—but that this patient must have got, at the hospitals to which he had applied for relief, mercurials, either alone or combined with purgatives. *If* that was the case, we have already an instance of the beneficial effects of chlorine," &c.—Now, we think, *if* Mr. Wallace will reconsider the whole of this evidence carefully, his own good sense will clearly convince him how very defective it must be considered by all those cautious spirits who take nothing for granted, and who have an instinctive horror of all suppositions.

As a mere matter of taste, we would also observe, that it is unusual to tack a Latin sentence at the commencement of each section of a work.

Mr. Wallace informs us that chlorine, applied to the surface of the body generally, or to the region of the liver in particular, has a decidedly beneficial effect upon the following complaints especially:—1°. Those diseases in which the liver is primarily affected. 2°. In diseases (probably of the liver), but in which the principal distress was referred to other parts, (including, indeed, almost every viscus or organ in the animal economy.) 3°. Diseases of which the liver was the principal seat, but which had been preceded by a morbid change of other parts. These sections are followed by remarks upon the actual effects of chlorine upon the skin, the mucous membranes, the respiration, and circulation, and on the brain and nerves. Besides its sensible effects, Mr. W. thinks it has an insensible operation, "a power of modifying, in a gradual manner, the tone of the organic fibre." Its principal sensible effects are, a pricking sensation of the skin, soreness of the mouth, and increased flow of saliva; and, locally, an eruption of minute papulæ.

To those who feel disposed to make trial of this remedy, the following information will be valuable. Chlorine may be used in a pure or diluted state; it may be diluted with atmospheric air only, or by this and aqueous vapour, and applied, generally or partially, at various temperatures, varying from 98° to 115°, or even 120°, Fahrenheit; when pure, it can only be used partially. Our author for a long time used RAPOU's instrument for DOUCHES, but now he employs a portable apparatus of his own invention, which may be placed in any room having a chimney: this is the machine of which the engraving is promised.

The following method of procuring the chlorine, and applying it locally, will conclude our notice of Mr. Wallace's work; and we have no doubt but that the remedy he has advocated will, under proper circumstances, prove a useful addition to our curative means.

The mode of preparing chlorine is as follows:—Muriate of soda and black oxyde of manganese are to be well triturated together, in the proportions of three of the former and one of the latter. Four parts of this powder are mixed with three parts of sulphuric acid, of the specific gravity of 1400 to 1000 of water; gentle heat is applied, and the gas is quickly brought over. A simple instrument for its partial application may be made by fixing a screw to a common cupping-glass, to which a stop-cock may be fitted; the glass is then applied to the part, and the air exhausted by the usual means: the gas is then introduced by means of the stop-cock, an assistant holding on the glass. Syringes for injecting the gas should not be made of brass.

Mr. Wallace will, we are quite sure, not be offended if we take our leave by suggesting to him the paraphrastic translation of *Ne quid nimis:* Do not ride your hobby-horse too hard.

